# High-quality genome of the zoophytophagous stink bug, *Nesidiocoris tenuis*, informs their food habit adaptation

**DOI:** 10.1093/g3journal/jkad289

**Published:** 2023-12-19

**Authors:** Tomofumi Shibata, Masami Shimoda, Tetsuya Kobayashi, Hiroshi Arai, Yuta Owashi, Takuya Uehara

**Affiliations:** Division of Insect Advanced Technology, Institute of Agrobiological Sciences, NARO, Tsukuba, Ibaraki 305-8634, Japan; Graduate School of Agricultural and Life Sciences, The University of Tokyo, Tokyo 113-8657, Japan; Graduate School of Agricultural and Life Sciences, The University of Tokyo, Tokyo 113-8657, Japan; Division of Insect Advanced Technology, Institute of Agrobiological Sciences, NARO, Tsukuba, Ibaraki 305-8634, Japan; Division of Insect Advanced Technology, Institute of Agrobiological Sciences, NARO, Tsukuba, Ibaraki 305-8634, Japan; Division of Insect Advanced Technology, Institute of Agrobiological Sciences, NARO, Tsukuba, Ibaraki 305-8634, Japan; Division of Insect Advanced Technology, Institute of Agrobiological Sciences, NARO, Tsukuba, Ibaraki 305-8634, Japan

**Keywords:** natural enemy, zoophytophagous, IPM, food habit

## Abstract

The zoophytophagous stink bug, *Nesidiocoris tenuis*, is a promising natural enemy of micro-pests such as whiteflies and thrips. This bug possesses both phytophagous and entomophagous food habits, enabling it to obtain nutrition from both plants and insects. This trait allows us to maintain its population density in agricultural fields by introducing insectary plants, even when the pest prey density is extremely low. However, if the bugs’ population becomes too dense, they can sometimes damage crop plants. This dual character seems to arise from the food preferences and chemosensation of this predator. To understand the genomic landscape of *N. tenuis*, we examined the whole genome sequence of a commercially available Japanese strain. We used long-read sequencing and Hi-C analysis to assemble the genome at the chromosomal level. We then conducted a comparative analysis of the genome with previously reported genomes of phytophagous and hematophagous stink bugs to focus on the genetic factors contributing to this species’ herbivorous and carnivorous tendencies. Our findings suggest that the gustatory gene set plays a pivotal role in adapting to food habits, making it a promising target for selective breeding. Furthermore, we identified the whole genomes of microorganisms symbiotic with this species through genomic analysis. We believe that our results shed light on the food habit adaptations of *N. tenuis* and will accelerate breeding efforts based on new breeding techniques for natural enemy insects, including genomics and genome editing.

## Introduction

Advancements in genome sequencing technology have recently facilitated whole genome sequencing of arthropods, including predatory insects that prey on agricultural pests (Li *et al.* 2019). However, numerous predatory insects possess minute body sizes and the maintenance of inbred lineages is challenging, resulting in considerable difficulty in obtaining highly contiguous genomes (Leung *et al.* 2020). By sequencing genomes with longer read length, more comprehensive gene annotation information and polymorphic mutation data can be acquired. Deciphering the whole genome of predatory insects is crucial for the development and selection of superior strains based on genomic information, thereby formulating effective breeding strategies for these natural enemies.

**Fig. 1.**
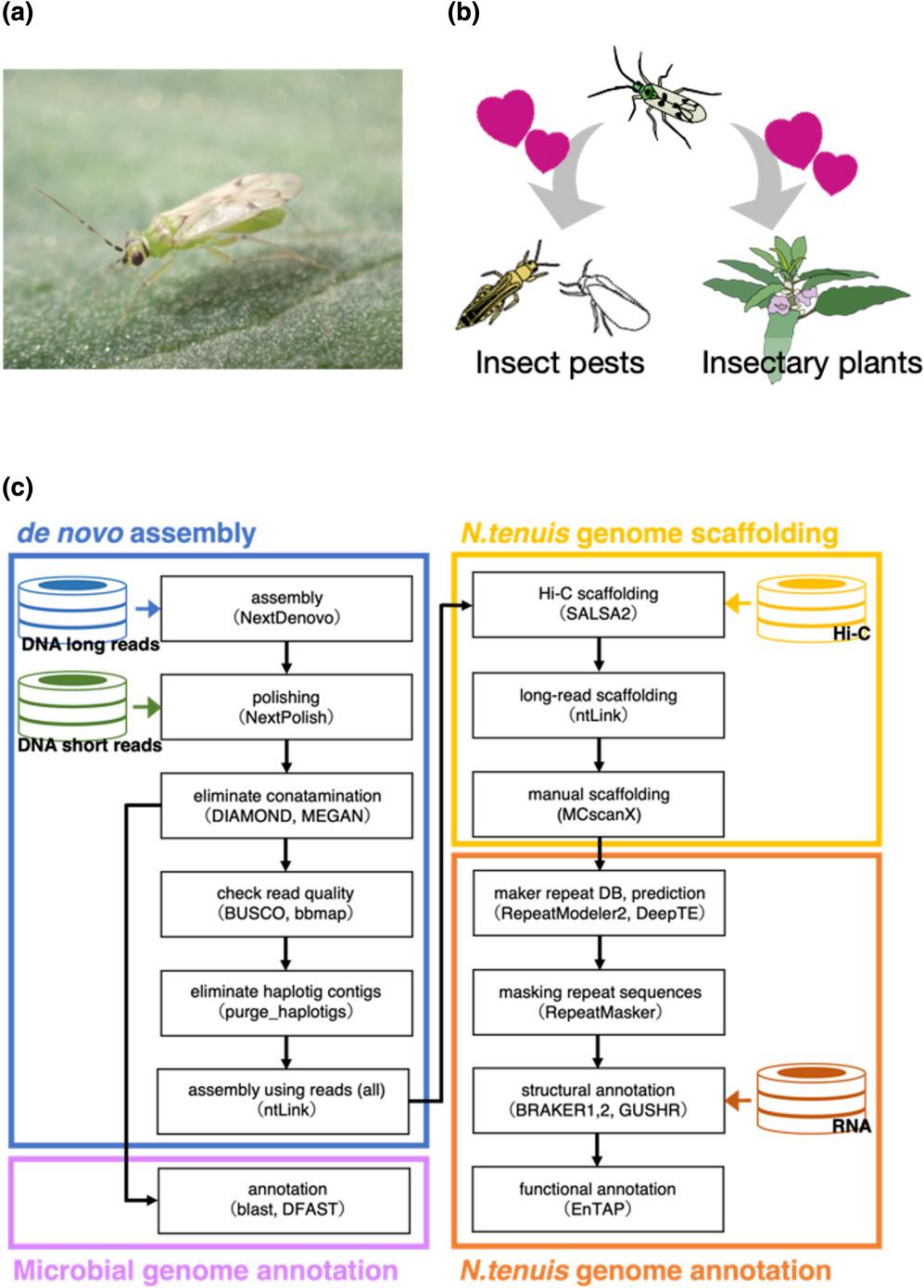
Overviews of the life history of *Nesidiocoris tenuis* and the pipeline used for genome analysis. a) Adult male of *Nesidiocoris tenuis* on a tomato leaf, b) schematic picture of the bug’s zoophytophagy, and c) pipeline of genome assembly and analysis. Cylindrical icons indicate read data and arrows show flow of processed data. The software used and the version in this pipeline are summarized in Supplementary Table 1.

*Nesidiocoris tenuis* (Reuter) (Fig. 1a) serves as a predatory insect across extensive regions from Asia to the Mediterranean (Pérez-Hedo and Urbaneja 2016; Yano 2022). This species is integral to the integrated pest management of micro-pests. Its predatory activity against pests that pose problems in greenhouse tomato cultivation, such as the silverleaf whitefly (*Bemisia tabaci*) and the tomato leafminer (*Tuta absoluta*), has garnered substantial interest (Urbaneja *et al.* 2005; Calvo *et al.* 2009; Sanchez *et al.* 2009; Itou *et al.* 2013). Owing to its zoophytophagous nature (Fig. 1b), *N. tenuis* can sustain its populations on plant-based food during periods of low pest infestation by incorporating insectary plants and is thus able to mitigate sudden pest outbreaks (Nakano *et al.* 2016, 2021). However, this insect’s strong preference for insectary plants complicates the control of its movement to crops; moreover, it can cause ring-shaped necrotic damage to tomatoes when the population density becomes excessive (Sanchez and Lacasa 2008; Calvo *et al.* 2009; Sanchez *et al.* 2009). This problem appears to originate from the bug’s feeding habits and chemosensory nature. However, research has focused primarily on behavioral observation (Chinchilla-Ramírez *et al.* 2020; Thomine *et al.* 2020), leaving the nature of the insect’s chemosensory receptors and neural substances largely unexplored.

Nevertheless, some pest management approaches that capitalize on these characteristics have been refined through various methods. For instance, the phototaxis of *N. tenuis* can be harnessed to effectively promote movement of the insects from insectary plants to tomato plants (Uehara *et al.* 2019b; Park and Lee 2021). Volatiles from infested plants (Rim *et al.* 2015, 2017, 2018, 2020) and conspecific individuals (Hall *et al.* 2021) attract *N. tenuis*. Thus, olfactory cues are also favorable candidates for promoting this bug’s movements. With respect to phytophagous feeding habits, research is under way to develop less aggressive strains through selective breeding (Chinchilla-Ramírez *et al.* 2020) and explore the potential of incorporating RNA interference with pest control strategies (Sarmah *et al.* 2021). Although a draft genome of this species was published by Ferguson *et al.* (2021) and the majority of genes have been annotated, further analysis of the genome structure is warranted. In Japan, several cryptic species of *N. tenuis* have been reported (Yasunaga 2017), necessitating the whole genome analysis of standard strains employed as biological control agents for the development and implementation of effective integrated pest management strategies.

Here, we investigated the whole genome of a Japanese commercial strain of *N. tenuis* using long-read sequencing and Hi-C analysis to assemble the genome at the chromosomal level (Fig. 1c). A comparative analysis of the genome with the previously reported genomes of phytophagous and hematophagous stink bugs was conducted to elucidate the genetic factors contributing to the herbivorous and carnivorous tendencies of this species. Furthermore, we report on the whole genomes of symbiotic microorganisms of this species identified through genomic analysis. From these results, we discuss feeding habit adaptation through the acquisition of chemosensory genes in *N. tenuis*.

## Materials and methods

### Insect

A commercial strain of *N. tenuis* (Fig. 1a) was obtained from Agrisect Inc. *Ephestia kuehniella* Zeller eggs (Ga-RanÒ; Agrisect Inc., Ibaraki, Japan) were used as food, with the eggs affixed to a 18mm×50mm label and provided ad libitum, and *Plectranthus amboinicus* (Lamiaceae) was used as an oviposition substrate and moisture source, with the leaves cut to approximately 8 cm in length. Bugs were maintained under $25\pm 1^{\circ }C$, 50–70% relative humidity, and a 16:8 light:dark photoperiod. To reduce heterozygosity, we crossed an unmated male and female, from Agrisect, placed them together in plastic cases (24×17×5cm), and established a sibling line by successive passages of the F1 generation. We used this line for the following sequencing analysis.

### Genomic DNA extraction

High-molecular-weight DNA was extracted using NucleoBond HMW DNA (MACHEREY-NAGEL GmbH & Co. KG, Düren, Germany) in accordance with the following manufacturers’ protocols. Eight hundred adult males of *N. tenuis* were separated into four groups of 200 individuals each, and each group was crushed with liquid nitrogen in a mortar and pestle until it became a fine powder, resulting in an average final DNA extract of approximately 60 ng/μl per sample with the protocol. Two hundred adult females were subjected to the same extraction method as the males. The amount of DNA extracted was adjusted to a total of 30 ng/μl, and this was filtered on the basis of length using a Short Read Eliminator Kit (Circulomics Inc., MD, USA) to remove any short DNA fragments. Additionally, for the Hi-C analysis, approximately 300 other males were processed in the Arima-HiC workflow for small animals, resulting in 30.5 ng/μl of DNA extract. A Nanodrop microvolume spectrophotometer (Thermo Fisher Scientific, MA, USA) and a Qubit 4.0 fluorometer (Thermo Fisher Scientific) were used for quality checks on each step of the DNA extraction.

### Library preparation and sequencing

Size-selected genomic DNA (gDNA) was ligated using a Ligation Sequencing Kit (SQK-LSK 112, Oxford Nanopore Technologies plc., Oxford, UK) and a NEBNext Companion Module for Oxford Nanopore Technologies Ligation Sequencing (New England Biolabs Inc., MA, USA). It was then purified with Agencourt AMPure XP beads (Beckman Coulter, CA, USA). We followed the protocol of the Ligation Sequencing Kit. One microgram of ligated DNA was sequenced on a flow cell (FLO-MIN112, Oxford Nanopore Technologies) using a MinION sequencer (Oxford Nanopore Technologies) to obtain raw sequence reads. Short-read sequencing was conducted using the NovaSeq 6000 platform, and the library was prepared in accordance with the manufacturer’s protocol (Illumina Inc.). For the Hi-C analysis, library was prepared by Arima-HiC (Arima Genomics, SD, USA) in accordance with the manufacturer’s protocol. The libraries were sequenced with a Novaseq6000 whole genome sequencing system.

### Assembly and polishing

The software used is listed in Supplementary Table 1. MinION raw data were base-called in high accuracy mode with Guppy v6.0.16. Sequence data were quality checked using Nanoplot v1.35.5 (De Coster *et al.* 2018). Genome size and heterozygosity were then estimated using k-mer analysis with GenomeScope 2.0 (Ranallo-Benavidez *et al.* 2020) and short-read paired-end sequences. The estimated genome size was used for genome size specification in the following analyses. Raw reads after quality checking were assembled using NextDenovo (https://github.com/Nextomics/NextDenovo) with an accurate setting (read_cutoff = 2k). The resulting contigs were polished using NextPolish v1.3.0 (Hu *et al.* 2020) with short-read paired-end sequence reads to correct for sequence errors. The resulting genome was verified using genome statistical values such as N50 (BBTools program stats.sh v44.0, https://jgi.doe.gov/data-and-tools/software-tools/bbtools/) and Benchmarking Universal Single-Copy Orthologue (BUSCO) v5.4.5 (Manni *et al.* 2021). Genome contamination from symbiotic microorganisms was removed by MEGAN and DIAMOND (Bagci *et al.* 2021). We lastly performed 1-kbp or less raw long-read assembly using ntLink with the “-gap_fill” option (Coombe *et al.* 2023).

### Scaffolding

Ligation regions of the Hi-C raw reads were removed using fastp v0.23.2 (Chen *et al.* 2018). Scaffolding was performed with the Hi-C reads using SALSA v2.3 (Ghurye *et al.* 2019). We next performed raw long-read-based scaffolding using ntLink (Coombe *et al.* 2023) and then finally checked manually for remaining gaps. We used GENESPACE (Wang *et al.* 2012) for the synteny comparison of scaffolded genomes and visualization of the results. The scaffolded genome was also used to create a contact map using nf-core/hic (Ewels *et al.* 2020) and visualized using HiGlass (Kerpedjiev *et al.* 2018).

### Structural and functional gene annotation

We identified repeat sequences using RepeatModeler v2.0.2a (Flynn *et al.* 2020) to increase the accuracy of the genome structural annotations. Estimated repeat regions were classified using DeepTE (Yan *et al.* 2020) and softmasked with RepeatMasker v4.1.2.p1 (Tarailo-Graovac and Chen 2009). The soft-masked genome was subjected to the gene prediction software BRAKER1 (Hoff *et al.* 2016) and BRAKER2 (Bruna *et al.* 2021). The predicted region was integrated using TSEBRA v1.0.3 (Gabriel *et al.* 2021), and then untranslated region sequences were added using GUSHR v1.0.0 (Keilwagen *et al.* 2016). For functional annotation, we used EnTAP v0.5.0 (Hart *et al.* 2020) with databases such as SwissProt or TrEMBL for invertebrates.

The genome data (contigs) of the symbiotic microbes were annotated with BLAST searches and DFAST (Tanizawa *et al.* 2017). Circularity of the contigs was confirmed using BLASTN searches, and each contig was closed manually as described by Arai *et al.* (2022).

### Phylogenetic and comparative analysis of genes

For phylogenetic analysis, we chose six hemipteran species with different feeding habits, namely *A. lucorum* (Liu *et al.* 2021), *O. laevigatus* (Bailey *et al.* 2022), *C. lectularius* (Rosenfeld *et al.* 2016), *P. micranthus* (Gäde and Marco 2022), *R. prolixus* (Mesquita *et al.* 2015), and *L. striatellus* (Zhu *et al.* 2017). We obtained the protein sequences of the six species using gffread v0.12.7 (Pertea and Pertea 2020) from genome (FASTA) and annotation (gene transfer format) data, and we removed pseudogenes using seqkit v2.3.1 (Shen *et al.* 2016). We then chose a single-copy core gene set in the BUSCO database (hemiptera_odb10) from the protein sequences and constructed an interspecific phylogenetic tree using iqtree v2.2.0.3 (Minh *et al.* 2020). The phylogenetic tree was converted to a tree considering divergence age using r8s v1.81 (Sanderson 2003). Genes orthologous among the six species were classified into orthogroups by OrthoFinder v2.5.4 (Emms and Kelly 2019). We estimated gene gain and loss rates using CAFE v4.2.1 (Han *et al.* 2013) from divergence, considering the phylogenetic tree and orthogroups.

We chose chemosensory receptors [gustatory receptor (GR), olfactory receptor (OR), ionotropic receptor (IR), pickpocket (PPK)], which have typical protein motif structures, from the Pfam database. Receptor genes were then aligned using mafft v7.508 (Nakamura *et al.* 2018), the alignment was trimmed with trimal v1.4.1 (Capella-Gutierrez *et al.* 2009), the phylogenetic tree was created with iqtree v2.2.0.3, and visualization was performed with Figtree v1.4.4 (https://github.com/rambaut/figtree).

The phylogenetic trees of the symbiotic microorganisms were drawn from the 16S rRNA gene inferred using the maximum likelihood method based on the Kimura two-parameter model with 1000 bootstrap replicates. Bootstrap values <60% are not shown and accession numbers are given after each operational taxonomic unit.

Cylindrical icons indicate read data and arrows show flow of processed data. The software used and the version in this pipeline are summarized in Supplementary Table 2.

## Results and discussion

### Genome size and heterozygosity estimates

Short-read sequencing male adults produced 50.6 Gb of data. The distribution of *k*-mers using k=21 showed high heterozygosity. We estimated a genome size of 275.9–278.3 Mb and a level of heterozygosity of 1.57–1.66% (Supplementary Fig. 1). The genome was about 50 Mb shorter than the previously reported one (Ferguson *et al.* 2021). In general, heterozygosity tends to be higher in wild insect strains and lower in inbred strains. Our heterozygosity result was very similar to previously reported genome (Ferguson *et al.* 2021) and not far from those revealed recently for other mirid species’ inbred strain genomes, namely that of *Cyrtorhinus lividipennis* (Bai *et al.* 2022) was 1.70%, of *Adelphocoris suturalis* (Xu *et al.* 2023) was 1.7%, and of *Apolygus lucorum* (Liu *et al.* 2021) was 1%.

### Genome sequencing and assembly

Before performing a genome assembly , we removed low-quality sequences (<Q8) and obtained approximately 29.6 Gb of raw reads (coverage=×110; Supplementary Fig. 2). The mean read length and N50 length of filtered reads were 3.4 kb and 11 kb, respectively. We performed de novo genome assembly with these reads, removed contamination and haplotigs from the assembled genome sequence (Fig. 1c), and finally obtained a draft genome with a size of 265.8 Mb. The draft genome had 92 contigs, an N50 of 6.5 Mb, and a GC content of 40%. The draft genome presented here is of high quality compared not only with those of mirid species with high heterozygosity but also with those of other insects with low heterozygosity (Lee *et al.* 2021; Chen *et al.* 2023; Stahlke *et al.* 2023). This ensures that our assembly meets the standards set by previous genome research.

Subsequently, we obtained a total of 101.5 Gb of raw data from the Hi-C library of the genome and 99.6 Gb of clean reads (without the adapter region sequence). Following chromosome scaffolding and manual curation, we obtained 72 contigs that anchored about 20 chromosomes (NTJ_v2.0, Table 1). We further selected 17 sequences, starting from the longest to the shortest, and compared their total length to the full genome length. We found that they covered 99.5% of the entire genome. This approach helped us to obtain a complete chromosome-level genome sequence (Supplementary Fig. 3). In fact, genomic in situ hybridization and comparative genomic hybridization analyses have shown that the karyotype of this species is 2n=32 (Ferguson *et al.* 2021). Our result is consistent with this finding. The chromosome-level genome consisted of 16.4 Mb of scaffold N50 and 41.7 Mb of maximum scaffold length. The BUSCO (Manni *et al.* 2021) of hemiptera_odb was 94.6% (single copy: 93.6%; duplicated: 1.0%). This chromosome-level assembly provided a substantial increase (>500-fold contiguity) in reference quality compared with the existing reference genome. We mapped raw sequence reads from females to the chromosome-level genome sequence, but there was no coverage bias in each contig; we were therefore unable to determine the sex chromosome.

**Table 1.** Summary statistics of our genomic analysis of *Nesidiocoris tenuis* (NTJ_v2.0) and comparison with the analysis of Ferguson *et al.* (2021): a) adult male of *Nesidiocoris tenuis* on a tomato leaf, b) schematic picture of the bug’s zoophytophagy, and c) pipeline of genome assembly and analysis.

	Genome size (Mb)	No. of contigs	No. of scaffolds	Scaffold N50 (Mb)	Scaffold N90 (Mb)	Max scaffold size (Mb)	BUSCO (%)
NTJ_v2.0	265.536	72	20	16.423	11.208	41.648	94.6
Ferguson_v1.5	355.120	51,852	36,513	0.287	0.033	1.392	87.9

**Fig. 2.**
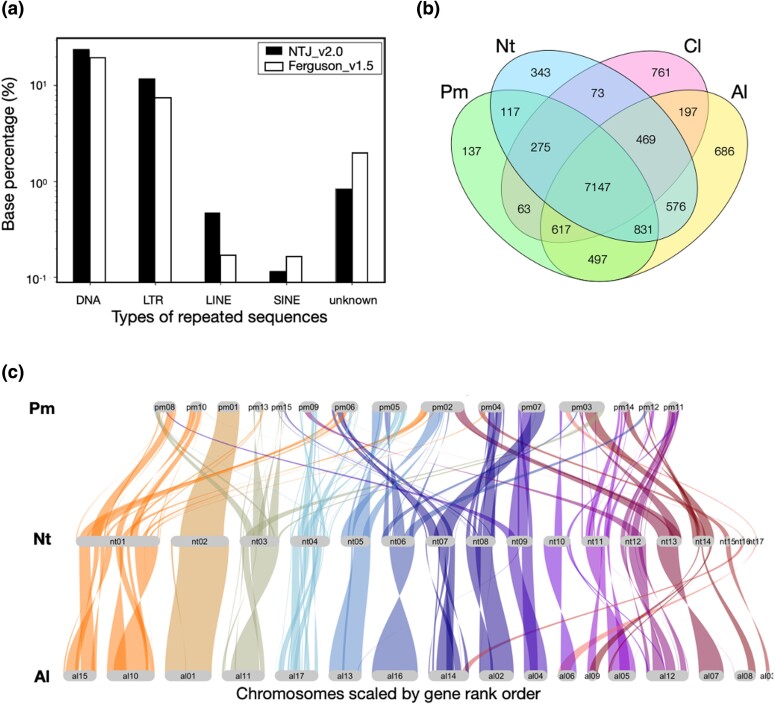
Comparisons of repeated sequences and genome landscapes in hemipteran chromosomes. a) Comparison of repeated sequences from this study and from that of Ferguson *et al.* (2021), b) Venn diagram analysis of all annotated genes in *Nesidiocoris tenuis* (Nt) with *Apolygus lucorum* (Al), *Pachypeltis micranthus* (Pm), and *Cimex lectularius* (Cl), and c) synteny analysis of Nt with Al and Pm.

The repeat sequence of this species was 95.9 Mb and about 36.1% of the whole genome sequence (Fig. 2a). This ratio is consistent with the ratio estimated from our *k*-mer frequency analysis using GenomeScope 2.0 (see Materials and Methods section). The ratios of repeat sequences were 23.7% for satellite DNA, 11.6% for long tandem repeats, 0.47% for long interspersed elements, and 0.12% for short interspersed elements. Compared with those of the previously reported genome (Ferguson *et al.* 2021), the long tandem repeat and long interspersed element sequences as a proportion of the whole genome sequences were increased, whereas the ratio of short interspersed elements was decreased. These results suggest that the continuity of the whole genome sequences was substantially improved.

### Comparative genome analysis of related species

The results of a Venn diagram analysis using the closely related species *A. lucorum* (Liu *et al.* 2021), *Pachypeltis micranthus* (Yang 2021), and *Cimex lectularius* (Rosenfeld *et al.* 2016) by OrthoVenn2 (Xu *et al.* 2019) showed that *N. tenuis* shared 9,023 orthologous genes with *A. lucorum* and 8,370 genes with *P. micranthus* (Fig. 2b). Additionally, we performed a synteny analysis of the genome NTJ_v2.0 and the chromosomal-level genomes from *A. lucorum* and *P. micranthus* (Fig. 2c). The results were consistent with the phylogenetic relatedness among these species. The regions of synteny were about 20% in common reciprocally, suggesting that our chromosomal genome was compatible with the quality of these genomes.

### Complete genomes of symbiotic microorganisms

Generally, antibiotic treatment or removal of the gut is performed to avoid contamination by symbiotic bacterial genomes. However, we were not able to perform this procedure because of the target species’ small body size and the large quantity of gDNA required for sequencing. Consequently, we unexpectedly determined the complete genome sequences of symbiotic microorganisms while compiling the draft genome of *N. tenuis*.

**Fig. 3.**
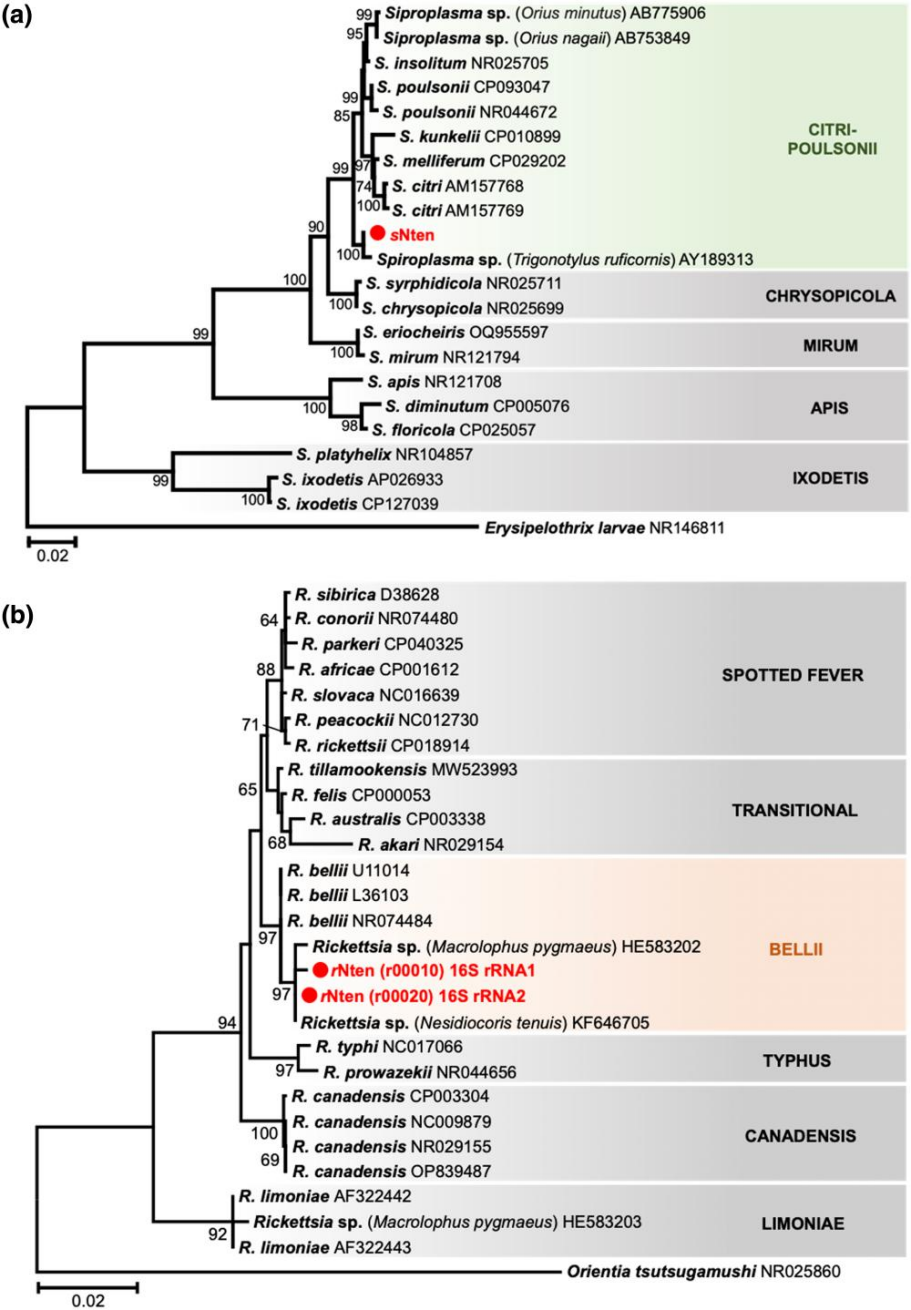
Phylogenetic analysis of symbiotic microorganisms. Phylogenetic trees based on the 16S rRNA genes of *Spiroplasma* and *Rickettsia* were inferred using the maximum likelihood method on the basis of the Kimura two-parameter model with 1,000 bootstrap replicates. Bootstrap values <60% are not shown, and accession numbers are given after each operational taxonomic unit. a) Phylogenetic tree of *Spiroplasma*, based on 1,477 positions of the 16S rRNA coding region. The classification of *Spiroplasma* is represented on the right side and grouped into the *citri–poulsonii* clade. The outgroup is *Erysipelothrix* larvae and the scale bar indicates 0.02 substitutions per site. b) Phylogenetic tree of *Rickettsia*, based on 1,402 positions of the 16S rRNA coding region. The classification of *Rickettsia* is represented on the right side and grouped into the *bellii* clade. The outgroup is *Orientia tsutsugamushi* and the scale bar indicates 0.02 substitutions per site.

On the basis of genome homologies, we confirmed that *N. tenuis* harbored both *Spiroplasma* (Molicutes) and *Rickettsia* (Alphaproteobacteria) endosymbionts, as previously described (Caspi-Fluger *et al.* 2014; Ferguson *et al.* 2021; Owashi *et al.* 2023). The *Spiroplasma* endosymbiont, referred to as the *s*Nten strain, has a main chromosome (1.5 Mb) with six plasmids (1,889 coding sequences in total) and was closely related to the *citri–poulsonii* group (Fig. 3a). The *Rickettsia* endosymbiont, referred to as the *r*Nten strain, had a main chromosome (2.3 Mb) with three plasmids (3,183 coding sequences in total) and was clustered into the *bellii* group (Fig. 3b).

*Spiroplasma* relatives of *S. citri* are frequently identified from hemipteran insects (Kwon *et al.* 1999; Watanabe *et al.* 2014) and from plants (Saglio *et al.* 1973; Yokomi *et al.* 2020). In the case of leafhoppers, *S. citri* is transmitted horizontally between plants and the insect (Mello *et al.* 2009). In addition, *Spiroplasma* endosymbionts sometimes manipulate their host’s reproduction selfishly in various ways. For example, some *Spiroplasma* strains clustered into the *Spiroplasma ixodetis* and *Spiroplasma poulsonii* groups induce male-killing in their host species, such as ladybirds and fruit flies (Montenegro *et al.* 2006; Tinsley and Majerus 2006). In addition, a *citri–poulsonii* group *Spiroplasma* strain induces male-killing in the green lacewing, *Mallada desjardinsi* (Hayashi *et al.* 2016).

The *Rickettsia* endosymbiont has been detected with high frequency in wild populations of *N. tenuis* in Israel (93–100%) and Japan (20.8–95.8%) (Caspi-Fluger *et al.* 2014; Owashi *et al.* 2023); this is consistent with our detection of *r*Nten in a commercial strain originating from the Japanese wild population. In fact, the nucleotide sequence of *r*Nten was identical to that of the 16S rRNA gene from the Israeli and Japanese populations. In the Israeli *N. tenuis*, *Rickettsia* were detected in the host gut lumen, suggesting that they may have a nutritional role in the host (Caspi-Fluger *et al.* 2014). However, the fact that some individuals of wild *N. tenuis* are not infected with *Rickettsia* (Caspi-Fluger *et al.* 2014; Owashi *et al.* 2023) means that infection is not obligatory for survival of the host. Some *Rickettsia* endosymbionts induce reproductive phenotypes such as male-killing (Lawson *et al.* 2001; von der Schulenburg *et al.* 2001) and parthenogenesis (Hagimori *et al.* 2006; Giorgini *et al.* 2010) in the host.

According to our genome and gene annotation (Supplementary Table 2), these genomes have genes encoding enzymes for oxidation or detoxification, besides genes for replicating themselves. We do not have enough data to discuss whether these genes contribute to the host’s feeding habits and survival; further experiments are needed to understand the impact of these symbiotic organisms.

### Chemosensory receptor genes involved in food preference

**Fig. 4.**
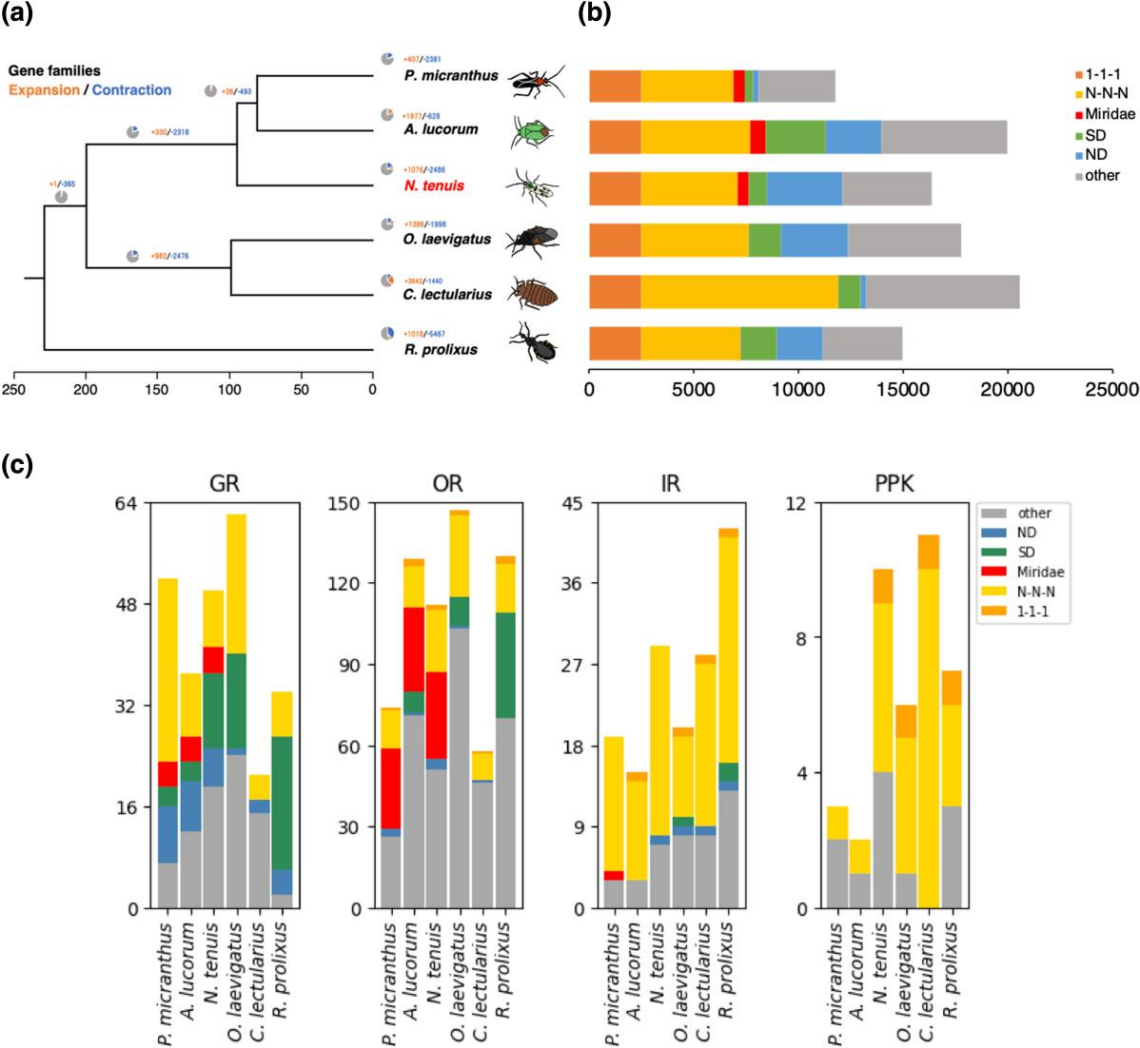
Comparison of annotated genes in closely related hemipteran species. a) Phylogenetic tree of closely related stink bugs and b) comparison of the number of annotated genes. Colors representing species in the left panel a) correspond to those in the right panel b). c) Comparison of chemosensory receptor genes. Abbreviations are as follows: ND, unclustered genes specific to each species; SD, species-specific genes with multiple gene copies; Miridae, Miridae-genus-specific genes; N-N-N, multicopy universal genes; 1-1-1, single-copy universal genes.

We constructed a phylogenetic tree with 1,286 common single-copy genes from 7 hemipteran species, namely *N. tenuis*, *A. lucorum*, *Orius laevigatus*, *C. lectularius*, *P. micranthus*, *Rhodnius prolixus*, and *Laodelphax striatellus*. We used *L. striatellus* as an outgroup (Fig. 4a). Unlike the other species, *N. tenuis*, *A. lucorum*, and *P. micranthus* belong to the same family, namely the Miridae. *Pachypeltis micranthus* is phytophagous, whereas *N. tenuis*, *A. lucorum*, and *O. laevigatus* are zoophytophagous. *C. lectularius, Rhodnius prolixus*, and *L. striatellus* are hematophagous. Examination of the phylogenetic tree showed that closely related species were clustered in the same clade, and *A. lucorum* and *P. micranthus* were located closer to each other than to *N. tenuis*. *Orius laevigatus*—a different genus but also zoophytophagous—was in a different clade from *N. tenuis*, and *R. prolixus* was in the farthest branch. This phylogenetic tree was based on common single-copy genes of these heteropteran insects and therefore reflected the closeness among the species. As mentioned earlier, our Venn diagram comparison of each species with all annotated genes showed that more genes in common were detected between closer species. These results suggest that feeding habits and life histories have diverged but that the ancestral gene sets have not changed greatly, irrespective of the feeding habit.

Divergence and convergence events in all annotated genes were analyzed using CAFÉ (Fig. 4a). We used the CAFÉ analysis to explore gene family expansion and contraction, and we estimated the *N. tenuis* gene birth rate at −0.0416108 with regard to duplications/gene/Mya. We detected 653 gene families that had experienced notable expansion or contraction events across the six species. The results demonstrated that genes had either diverged or converged with speciation, yet we found no universal patterns that mirrored phylogenetic relationships or dietary habits.

**Fig. 5.**
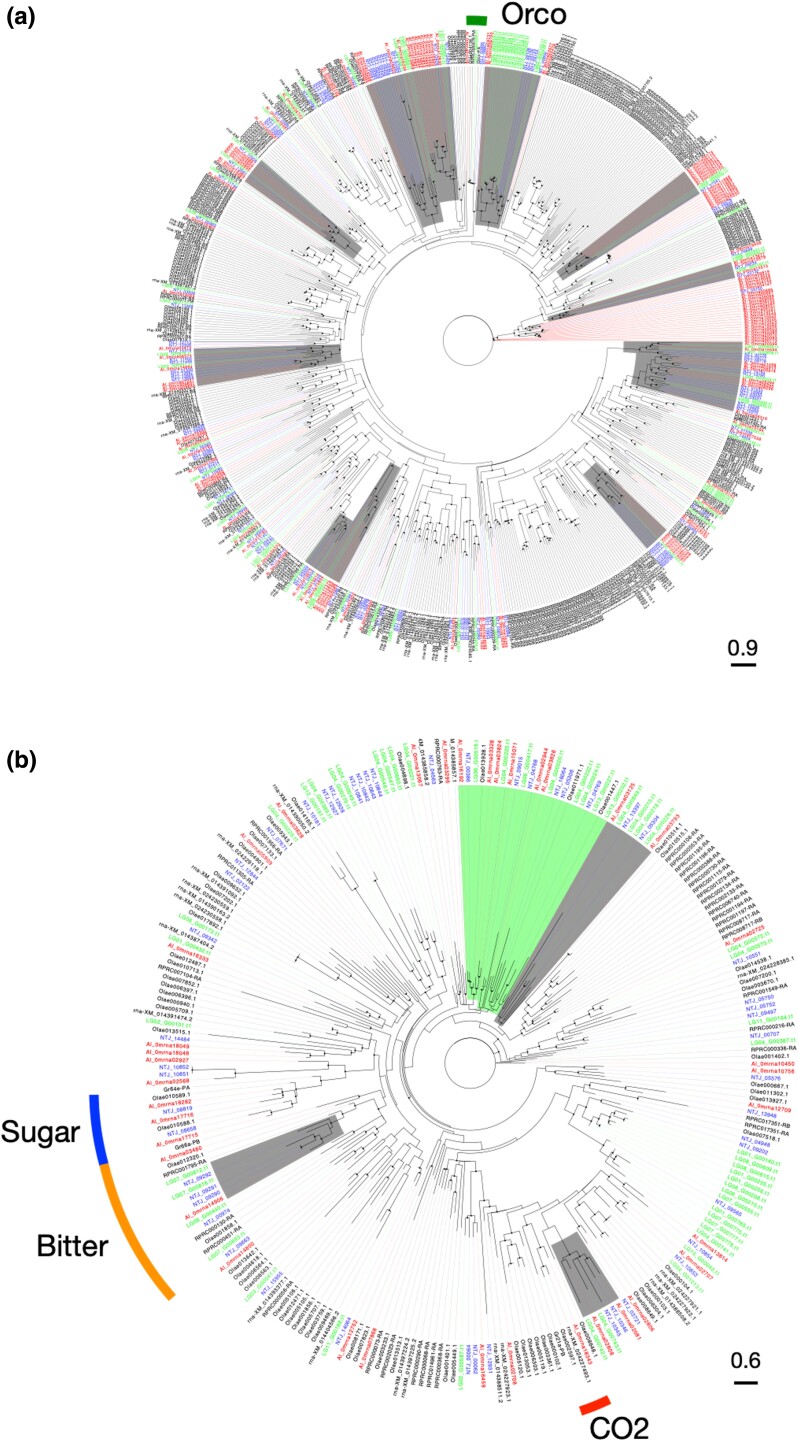
Phylogenetic analysis of chemosensory genes. Phylogenetic trees of a) olfactory and b) gustatory genes. Black circles indicate branches with bootstrap values of 80% or higher. The gray highlights indicate a clade consisting of three species from Miridae (*Apolygus lucorum*, *Nesidiocoris tenuis*, and *Pachypeltis micranthus*), while the green highlights represent a clade comprising four species with herbivorous feeding habits (*A. lucorum*, *N. tenuis*, *P. micranthus*, and *Orius laevigatus*). Each clade is characterized by the presence of at least five genes. Commonly found insect chemosensory receptor genes, such as olfactory coreceptor (*Orco*) and sweet, bitter, and ${\text{CO}}_{2}$ GRs are shown.

We next focused on genes associated with feeding habit, such as chemosensory receptor genes. As chemosensory genes, we focused on OR, GR, IR, and PPK of the degenerin/epithelial sodium channel gene family. Chemosensory genes commonly identified in insects, such as olfactory coreceptor (*Orco*), and GRs for sugar, bitter, and ${\text{CO}}_{2}$ sensation were revealed, but we currently lack ligand information or the relevant literature concerning other ORs or GRs. Comparison of gene counts showed characteristic differences between ORs and GRs, while no notable disparities were found in IR and PPK (Fig. 4b,c). For instance, the comparison of OR genes indicated the presence of those specific to Miridae, whereas that of GR genes indicated the existence of those that were more species specific. In fact, our phylogenetic analysis of OR genes frequently revealed mirid-specific and non-mirid-specific OR gene clades throughout the tree. One plausible interpretation for this finding is that ancestral mirid species procured a particular set of OR genes at a certain juncture and, since then, they have utilized similar olfactory signals with a comparable set of ORs (Fig. 5a). While OR gene clades reflect phylogenetic relation, these do not align with their food habits. It seems unlikely that ORs play a central role in the adaptation of feeding behaviors. This is consistent with the transition scenario from zoophytophagous to phytophagous habits proposed by Wheeler (2001).

In contrast, in the case of GR genes, mirid-specific clusters as large as the OR gene clusters were not observed, but were instead identified as small clusters (Fig. 5b). Notwithstanding, the herbivore-specific GR genes formed a clade (Fig. 5b). These findings suggest that some GRs are common among herbivorous species and may detect similar plant chemicals. However, each species appears to lean more toward acquiring its own specific GRs in alignment with its dietary habits. In terms of the different effective ranges of signals detected by olfaction and gustation, olfactory and gustatory cues generally provide signals for long and close distance, respectively (Bruce *et al.* 2005). Plants are immobile organisms, so that a combination of these two sensory signals is crucial for finding a host, particularly in herbivorous species (Silva and Clarke 2020). For example, in lepidopteran insects, the egg-laying preference of female adults determines the larval feeding habitat and survival. It has been suggested that expansion of the availability of the preferred host (i.e. extension of the range of detection of plant secondary metabolites) is linked with the presence of duplicating taste receptors (Engsontia *et al.* 2014; Suzuki *et al.* 2018). Our results indicated that the number of GRs was almost identical in zoophytophagous and phytophagous species, yet higher than in hematophagous species. This variation can potentially be elucidated by comparing the diversity of ligand molecules required for detecting animal- and plant-based foods, alongside an examination of the respective nutritional values of each food.

### Future perspectives on breeding natural enemies using genomics and genome editing

Biological control agents, such as natural enemy insects, represent pest management solutions that incur a small environmental burden and reduce labor intensity. However, as living organisms, these insects may not always exhibit the targeted effectiveness of chemical pesticides. We have developed tools to address this issue thus far (Ogino *et al.* 2016; Tokushima *et al.* 2016; Uehara *et al.* 2019a, 2019b). With the advent of genome editing technology, enhancement of the efficacy of natural enemies through breeding has become increasingly feasible. In this context, we have constructed a highly contiguous genome that can serve not only as a reference sequence for genome editing but also for the analysis of variants in, for example, single nucleotide polymorphisms in field populations. We believe that the genome revealed here will contribute to explorations of the biological functions and beneficial traits of *N. tenuis*.

## Data Availability

All raw reads obtained for genome assembly have been deposited in the DNA Data Bank of Japan under BioProjectID PRJDB16217 and Sequence Read Archive (SRA) under DRR495435. Supplemental materials are available on figshare: https://doi.org/10.6084/m9.figshare.24212835.v1. Supplemental material is available at GTHREE online.
